# ATM/p53-Associated Apoptosis Contributes to Sterility in Allotriploid Crucian Carp

**DOI:** 10.3390/ani16192990

**Published:** 2026-09-23

**Authors:** Jing Wang, Xinyu Xie, Yan Wang, Mengli Li, Jiahao Wu, Yu Deng, Zhongyuan Shen, Kaikun Luo

**Affiliations:** 1 Engineering Research Center of Polyploid Fish Reproduction and Breeding of the State Education Ministry, College of Life Sciences, Hunan Normal University, Changsha 410081, China; 2 Yuelushan Laboratory, Changsha 410128, China

**Keywords:** allotriploid crucian carp, sterility, ATM/p53, apoptosis, meiosis, pachytene checkpoint

## Abstract

During meiosis, ataxia telangiectasia mutated (ATM) kinase senses DNA double-strand breaks (DSBs) and regulates multiple downstream processes, including DSB repair and p53-mediated apoptosis. The allotriploid crucian carp (3nAT, 3n = 150), derived from distant hybridization between female diploid red crucian carp (2nRCC) and male allotetraploid hybrids (4nAT), is sterile and exhibits extensive germ cell apoptosis associated with abnormal meiosis. Here, we used the ATM kinase inhibitor KU55933, to investigate the sterility mechanism. Following intraperitoneal treatment with KU55933 (KU-3nAT), germ cell apoptosis was markedly reduced, and a small number of morphologically abnormal gametes were produced. Meiosis- and gamete maturation-related genes were upregulated, whereas pro-apoptotic genes were downregulated, indicating that meiotic arrest was partially attenuated. However, KU-3nAT sperm exhibited abnormal structures with rounded heads, retained cytoplasm, and absent or malformed flagella. Self-crossed and hybrid embryos with 2nRCC arrested at gastrulation, while a few KU-3nAT ♀ × 4nAT ♂ embryos hatched. These results suggest that ATM/p53-associated apoptosis contributes to germ cell loss at the proposed pachytene checkpoint in 3nAT, while ATM inhibition permits limited gamete production but does not restore normal gamete function or embryonic development.

## Simple Summary

The allotriploid crucian carp (3nAT) is a sterile hybrid fish containing three sets of chromosomes. This study investigated why these fish are unable to produce viable offspring. Using the ATM kinase inhibitor KU55933, we found that germ cell apoptosis associated with abnormalities in meiotic chromosome pairing in 3nAT is accompanied by activation of the ATM/p53 signaling pathway. When ATM was inhibited, germ cell death was reduced and some abnormal sperm and eggs were produced, but these gametes were still incapable of supporting normal embryonic development. The findings suggest that ATM/p53-associated surveillance contributes to germ cell loss and sterility in 3nAT and provide new insights into the molecular basis of reproductive failure in polyploid fish.

## 1. Introduction

In sexually reproducing organisms, the strict molecular regulation of meiosis is essential for normal gamete formation and genetic stability. During meiotic prophase I, DNA double-strand breaks (DSBs) are introduced by SPO11 and other factors after DNA replication. ATM and ATR kinases sense DNA damage, activate the stress response system, and induce cell cycle arrest to either complete DNA repair or initiate apoptosis [1,2,3,4]. It has also been reported that ATM primarily responds to double-stranded DNA breaks, while ATR is activated primarily by RPA-coated ssDNA arising during DNA end resection [5].

When ATM receives DNA damage signals, it phosphorylates downstream effectors including p53, BRCA1/BRCA2, and NBS1. Among these, p53 was one of the first identified ATM targets. p53-dependent cellular outputs following DNA-damage stimuli are multifaceted and context-dependent. In certain biological contexts, p53 can promote cell-cycle arrest to facilitate DNA repair or drive apoptosis via transcriptional up-regulation of pro-apoptotic factors (e.g., Bax, Bad, IGF-BP3) and repression of anti-apoptotic Bcl-2 [6,7,8].

Given that ATM/p53-mediated apoptosis functions as a DNA damage surveillance mechanism during meiosis, an important question arises: does this pathway also underlie the meiotic failure observed in polyploid fish, where chromosome set imbalance may disrupt homologous pairing? Reproductive disorders in triploid fish have been extensively documented, including altered gonadal structure in triploid Indian catfish [9], inhibited spermatogenesis in triploid sea bass [10], and reduced testis volume in triploid gilthead seabream and yellowtail flounder [11,12]. Studies have primarily focused on histology [9,13], neuroendocrine regulation [14,15], and transcriptomics [16,17]. However, the molecular mechanisms underlying meiotic arrest, particularly the surveillance of homologous synapsis and recombination, remain poorly understood.

The allotriploid crucian carp (3nAT, 3n = 150) was produced by crossing female diploid red crucian carp (*Carassius auratus* red var., 2nRCC, 2n = 100) with male allotetraploid hybrids (4nAT, 4n = 200), which were obtained through multiple generations of distant crossing between red crucian carp (♀) and common carp (*Cyprinus carpio*, ♂) [18,19]. Cytogenetic and histological characterization has established that 3nAT carries 3n = 150 chromosomes, comprising two sets of crucian carp chromosomes and one set of common carp chromosomes, and that its gonads are severely degenerated, with ovaries arrested before stage II and testes lacking normal spermatozoa [19,20,21]. On the basis of these established observations, we hypothesized that the single set of common carp chromosomes cannot complete synaptonemal pairing during meiotic prophase I, potentially leaving persistent DSBs that may activate ATM/p53-mediated apoptosis at the pachytene checkpoint and thereby may contribute to germ cell apoptosis and sterility. Furthermore, sex hormone rescue experiments showed that although exogenous hormones promoted gamete maturation-related gene expression and enabled the production of abnormal gametes, the treated 3nAT remained sterile, indicating that sex hormones affect gamete maturation but are not the determinant of triploid sterility [22].

The pachytene checkpoint is a meiosis-specific surveillance mechanism that monitors homologous chromosome recombination and synapsis [23]. When recombination and synapsis are defective, the checkpoint is activated to arrest the cell cycle at the pachytene stage. If defects remain uncorrected, downstream responses vary across species from cell cycle delay in yeast to p53-mediated apoptosis in mammals [24,25]. Although defective synapsis has been linked to p53-dependent germ cell apoptosis in a *Dmc1*-knockout medaka model, the ATM/p53-mediated apoptotic pathway downstream of the pachytene checkpoint has not been directly demonstrated in allotriploid fish.

KU-55933 is a potent, selective ATM kinase inhibitor originally characterized in human HeLa cells, where it abrogates ionizing-radiation-induced ATM-dependent phosphorylation of p53, NBS1 and Chk2 [26]. The downstream outcomes of ATM inhibition are context-dependent: it attenuates ATM/p53-dependent mitochondrial apoptosis induced by 2-hydroxyethyl methacrylate in human dental pulp cells [27] and by microcystin-LR in mouse spermatogonial GC-1 cells [28]. However, phenotypic alterations resulting from KU55933-mediated ATM inhibition alone cannot directly confirm downstream p53 activation in our fish model. Either genetic validation or protein-level detection of phosphorylated signaling intermediates would be required to definitively establish the full ATM-p53 signaling cascade.

In 3nAT, whether the inability to perform homologous recombination repair after DSBs in common carp-derived chromosomes leads to ATM-mediated p53 activation and subsequent germ cell apoptosis provides a valuable model for studying allotriploid sterility. Here, we hypothesized that synapsis failure of common carp-derived chromosomes is associated with activation of ATM/p53-mediated apoptosis at the proposed pachytene checkpoint and contributes to 3nAT sterility. To test this, we used the ATM inhibitor KU55933 and examined changes in gonadal structure, germ cell apoptosis, meiotic gene expression, gamete production, and fertilization capacity.

## 2. Materials and Methods

### 2.1. Ethics Statement

All experiments were performed in accordance with the Guidelines for the Care and Use of Laboratory Animals and were approved by the Animal Care Committee of Hunan Normal University (Changsha, China; Approval No. 20251045). All fish were deeply anesthetized with 100 mg/L MS-222 (Sigma-Aldrich, St. Louis, MO, USA) before dissection, and all efforts were made to minimize suffering.

### 2.2. Experimental Fish

Allotriploid crucian carp (3nAT, 3n = 150), diploid red crucian carp (2nRCC, 2n = 100) and male allotetraploid crucian carp common carp hybrids (4nAT, 4n = 200)were obtained from ponds at the Engineering Research Center of Polyploid Fish Breeding of the State Education Ministry, Hunan Normal University (Changsha, China). The ploidy levels of all experimental fish were confirmed by flow cytometry (Partec, now Sysmex Partec GmbH), Görlitz, Germany) prior to experimentation. Fish were cultured in open ponds with suitable water temperature (18–24 °C), pH (7.0–8.5), and dissolved oxygen (5.0–8.0 mg/L), and were fed with commercial formulated feed. Sex was determined based on secondary sexual characteristics: males had long, narrow pectoral fins with a conical keratinized cuticle protruding from the first fin ray, rough body surface, and a slim abdomen; females had short, fan-shaped pectoral fins with smooth first fin rays, an enlarged abdomen with bright red gonopore, and a smoother body surface.

### 2.3. KU55933 Treatment

KU55933 (CAS No. 587871-26-9, MedChemExpress, Monmouth Junction, NJ, USA) was dissolved in dimethyl sulfoxide (DMSO) to prepare a 10 mM stock solution, which was diluted in physiological saline immediately before use. To determine the in vivo working concentration, a preliminary dose-ranging experiment was first performed in 3nAT using 1, 5, 10, 20, 50, 100, and 200 μM solutions injected intraperitoneally at 0.5 mL/100 g body weight; 10 μM was selected as it alleviated germ-cell apoptotic phenotypes in exploratory observations while the fish remained physiologically normal. In the formal experiment, 3nAT (body weight 200–300 g; *n* = 40 per group) at 4 months of age, when meiosis has already been initiated, were intraperitoneally injected with 10 μM KU55933 at 0.5 mL/100 g body weight, corresponding to 50 nmol/kg (approximately 20 μg/kg) KU55933 per injection, every 48 h for seven consecutive injections. Because no pharmacokinetic data are available for KU55933 in fish, this repeated-injection schedule was used to maintain ATM inhibition over the treatment window, based on the short plasma half-life reported in mammals. The vehicle-treated 3nAT control group received equivalent volumes of vehicle (physiological saline containing 0.1% DMSO) on the same schedule. Of note, repeated-injection handling was only performed for 3nAT fish in this experiment; 2nRCC individuals were maintained under identical pond-rearing conditions but did not receive any injection treatment. No mortality or overt adverse effects were observed during the seven injections. After the injection regimen, the fish were continuously cultured in ponds until the breeding season.

### 2.4. Histological Observation of Gonads

Gonadal tissues from 2nRCC, 3nAT, and KU-3nAT (five males and five females per group) were dissected during the breeding season. For paraffin sections, gonadal tissues were fixed in Bouin’s solution overnight, dehydrated through a graded ethanol series, cleared in xylene, and embedded in paraffin. Serial sections (6–8 μm) were cut using a microtome (RM2015, Leica, Wetzlar, Germany) and stained with hematoxylin and eosin (H&E). The microstructure of gonadal tissues was observed and photographed under a light microscope (CKX41-32PH, Olympus, Tokyo, Japan).

For ultrastructural observation, gonadal tissues (2 mm × 2 mm × 2 mm) were fixed in 2.5% glutaraldehyde, post-fixed in osmic acid, dehydrated through a graded acetone series, and embedded in Epon812 (Sigma-Aldrich, Burlington, MA, USA). Ultrathin sections (60 nm) were cut using an ultramicrotome (EM UC7, Leica, Wetzlar, Germany) and stained with uranyl acetate and lead citrate. The ultrastructure was observed using a transmission electron microscope (HT7800, Hitachi, Tokyo, Japan).

### 2.5. Sperm Morphology Analysis

Sperm from KU-3nAT and 2nRCC males were collected by manually squeezing the abdomen after artificial induction (detailed hormone-injection procedures are described in Section 2.8). Sperm morphology was analyzed using multiple staining and imaging methods.

Papanicolaou staining: Sperm smears were fixed and stained using the Papanicolaou staining method to observe the head, neck, and flagellum structure.

Aniline blue-eosin staining: Sperm smears were stained with aniline blue-eosin to assess sperm maturity. Mature sperm were stained orange, whereas immature sperm were stained blue.

Scanning electron microscopy (SEM): Sperm were fixed in 2.5% glutaraldehyde, dehydrated through a graded ethanol series, critical-point dried, sputter-coated with gold, and observed under a scanning electron microscope (SM-6360LV, JEOL, Tokyo, Japan).

Transmission electron microscopy (TEM): Sperm were fixed and processed as described in Section 2.4 for ultrastructural observation.

Immunofluorescence: Sperm were fixed in 4% paraformaldehyde (PFA), permeabilized with 0.1% Triton X-100, and incubated overnight at 4 °C with anti-α-tubulin (ab7291, Abcam, Cambridge, MA, USA) and anti-γ-tubulin (ab179503, Abcam) primary antibodies. Samples were then incubated with DyLight 488-conjugated goat anti-rabbit IgG (A23220, Abbkine, Wuhan, China) and DyLight 594-conjugated goat anti-mouse IgG (A23410, Abbkine) secondary antibodies at 37 °C for 1 h, followed by DAPI staining for 5 min. Sperm structure was observed and imaged using a laser scanning confocal microscope (FV1200, Olympus, Tokyo, Japan).

### 2.6. TUNEL Assay

Germ cell apoptosis in gonadal tissues of 3nAT and KU-3nAT was detected using the In Situ Cell Death Detection Kit, TMR red (Roche, Basel, Switzerland). Gonadal tissues were fixed in 4% PFA overnight, dehydrated in 30% sucrose, embedded in Optimal Cutting Temperature compound (O.C.T., Sakura, Tokyo, Japan), and sectioned at 5 μm. After fixation in 4% PFA for 15 min, sections were washed, blocked in glycine-HCl solution, and permeabilized with 0.1% Triton X-100 in citrate solution. TUNEL reaction mixture was applied and incubated at 37 °C for 60 min. DAPI was used for counterstaining. Apoptotic signals (red fluorescence) were observed using a laser scanning confocal microscope (FV1200, Olympus) at an excitation wavelength of 590 nm.

For TUNEL analysis, three non-overlapping tissue sections were selected for each individual fish, and five representative random fields were captured per section. Image assessment was conducted under blinded conditions to reduce subjective observer bias. Formal signal normalization was not performed owing to inherent heterogeneity across gonadal sections. It should be noted that our TUNEL evaluation is primarily a qualitative cytological observation, rather than a strict quantitative apoptotic-index measurement.

### 2.7. RNA Isolation and Quantitative Real-Time PCR (qPCR)

Total RNA was extracted from gonadal tissues (testis and ovary) and female liver from 3nAT, and KU-3nAT using TRIzol Reagent (Invitrogen, Carlsbad, CA, USA). Three biological replicates were collected for each tissue group (these fish were a subset of the animals used for histology). The amount and purity of RNA were determined by spectrophotometry and agarose gel electrophoresis. After treatment with RNase-free DNase (Promega, Madison, WI, USA), 1 μg total RNA was reverse-transcribed into complementary DNA (cDNA) using PrimeScript^TM^ II 1st Strand cDNA (Takara Biotechnology, Dalian, China) with random primers.

qPCR was performed on a QuantStudio 5 Real-Time PCR System (Applied Biosystems, Foster City, CA, USA) using PowerUp SYBR Green Master Mix (Applied Biosystems). The reaction mixture (10 μL) contained 2.5 μL cDNA, 5 μL SYBR Green Master Mix, 0.5 μL forward primer, 0.5 μL reverse primer, and 1.5 μL nuclease-free water. The amplification conditions were 50 °C for 5 min and 95 °C for 10 min, followed by 40 cycles of 95 °C for 15 s and 60 °C for 45 s. Each sample was analyzed in three technical replicates, and each plate included a no-template control (NTC) and a no-reverse-transcriptase control (NRT). *β-actin* was used as the reference gene; its stability under the present conditions (gonadal tissues and KU55933 treatment) was verified in preliminary experiments, in which its Cq values remained stable across samples. The genes analyzed included: meiosis-related genes (*spo11*, *rad51*, *dmc1*, *rec8*, *scp1*, *atm*), pro-apoptotic genes (*p53*, *bad*, *bid*), sperm maturation-related genes (*spef2*, *rnf8*, *ift27*, *tekt1*, *piwil2*), and the vitellogenesis gene (*vtg*). The primers used in this study are listed in Table 1. The expression levels of target genes were normalized to *β-actin* using the 2^−ΔΔCt^ method. A melting curve analysis was performed to validate specific amplification.

### 2.8. Artificial Reproduction and Embryo Development Observation

During the breeding season, KU-3nAT fish were artificially induced by intraperitoneal injection of luteinizing hormone-releasing hormone analogue (LHRH-A2) at 5 μg/kg and human chorionic gonadotropin (HCG) at 400 IU/kg for females, and 2.5 μg/kg LHRH-A2 and 200 IU/kg HCG for males. Five crossing groups were established: (1) KU-3nAT ♀ × KU-3nAT ♂ (intra-group cross), (2) KU-3nAT ♀ × 2nRCC ♂, (3) 2nRCC ♀ × KU-3nAT ♂, (4) KU-3nAT ♀ × 4nAT ♂ and (5) 2nRCC intra-group cross as the control (each group contained >2000 fertilized eggs). Fertilized eggs were cultured in dishes filled with water at 19–20 °C. The key processes of embryogenesis—including zygote, 1-cell, morula, blastula, gastrula, late gastrula, tail bud, somite stages, and fry—were observed and photographed under a stereoscope (MZ16FA, Leica).

### 2.9. Statistical Analysis

Data are presented as mean ± S.E.M. Biological replicates (*n* = 3 independent fish per group) were analyzed separately, and technical replicates were averaged within each biological replicate before statistical testing. Differences among groups were assessed by one-way ANOVA followed by Tukey’s post hoc test for multiple pairwise comparisons (or Dunnett’s post hoc test when each group was compared against a single control), using GraphPad Prism 7.04. *p* < 0.05 was considered statistically significant, and the statistical test used and significance thresholds are indicated in the figure legends.

## 3. Results

### 3.1. Changes in Gonad Microstructure After KU55933 Treatment

After intraperitoneal injection with KU55933, the microstructure of both ovaries and testes of KU-3nAT exhibited obvious changes compared with untreated 3nAT (Figure 1). In the ovaries, 2nRCC controls contained many oocytes at phase II (Figure 1A,B), whereas no apparent phase II oocytes were detected in 3nAT ovaries (Figure 1E,F). In KU-3nAT ovaries, some phase II oocytes were observed (Figure 1I,J; black arrows). In the testes, 2nRCC contained a large number of mature sperm with rounded, darkly stained head nuclei (Figure 1C,D). In 3nAT, sperm cells showed abnormal nuclear condensation and irregular morphology (Figure 1G,H). In KU-3nAT testes, seminiferous lobule-like cavities containing a small number of sperm were observed (Figure 1K,L; black arrows).

### 3.2. Structural Features of Sperm

Sperm produced by KU-3nAT males during the breeding season were further analyzed using Papanicolaou staining, aniline blue-eosin staining, immunofluorescence, and electron microscopy (Figure 2 and Figure 3).

Under Papanicolaou staining, the normal sperm of 2nRCC possessed a distinct head, neck, and flagellum (Figure 2A). In contrast, the sperm of KU-3nAT had round heads with condensed chromatin but retained surrounding cytoplasm (Figure 2B). Furthermore, KU-3nAT sperm lacked a distinct flagellum. Aniline blue-eosin staining showed that 2nRCC sperm were stained rose-pink, indicating maturity (Figure 2C), while KU-3nAT sperm were stained blue, indicating immaturity (Figure 2D). Immunofluorescence staining with anti-α-tubulin and anti-γ-tubulin antibodies revealed that KU-3nAT sperm had abnormal flagellar and neck structures (Figure 2E).

Scanning electron microscopy (SEM) showed that 2nRCC sperm had normal morphology with a smooth round head, intact neck, and flagellar structure (Figure 3A). KU-3nAT sperm exhibited folds on the head surface (Figure 3B), and the majority lacked flagellar structure; a few sperm possessed multiple flagella (Figure 3C). Transmission electron microscopy (TEM) observation revealed that KU-3nAT sperm heads contained condensed chromatin of varying sizes (Figure 3D), and some sperm nuclei were crumpled, exhibiting morphology resembling apoptotic bodies (Figure 3E). The 9 + 2 axonemal structure of the flagellum was observed in a few KU-3nAT sperm (Figure 3F).

### 3.3. Germ Cell Apoptosis in the Gonads

The developmental fate of germ cells in the gonads of 3nAT was examined by TUNEL assay. Prominent apoptotic signals (red fluorescence) were detected in both the testes and ovaries of untreated 3nAT, whereas fewer TUNEL-positive signals were observed in KU-3nAT following KU55933 treatment (Figure 4).

### 3.4. Expression Changes in Germ Cell Development Genes After KU55933 Treatment

To further investigate the changes in the expression profiles of germ cell development genes after KU55933 treatment, we characterized the expression of genes associated with homologous recombination (*spo11*, *rad51*, *dmc1*), synapsis (*rec8*, *scp1*), and the key checkpoint factor *atm* in both ovaries and testes, and sperm maturation-related genes (*spef2*, *rnf8*, *ift27*, *tekt1*, *piwil2*) in testes and the vitellogenesis gene (*vtg*) in female liver.

After KU55933 treatment, the expression levels of *spo11*, *rad51*, *dmc1*, *rec8*, and *scp1* in both the ovaries and testes of KU-3nAT were significantly higher than those in 3nAT (*p* < 0.05; Figure 5A–D). In contrast, the expression level of *atm* was significantly lower in KU-3nAT gonads than that in 3nAT.

The expression levels of *spef2*, *rnf8*, *ift27*, *tekt1*, and *piwil2* were significantly higher in KU-3nAT testes compared to 3nAT (*p* < 0.05; Figure 5E). The *vtg* gene was significantly upregulated in the liver of female KU-3nAT compared to both 3nAT and 2nRCC females (*p* < 0.05; Figure 5F).

Consistent with the TUNEL results, the expression levels of pro-apoptotic genes were significantly downregulated in KU-3nAT. The mRNA expression levels of *p53*, *bad* and *bid* were all significantly lower in KU-3nAT gonads than those in 3nAT (*p* < 0.05; Figure 5G,H).

### 3.5. Fertilization and Embryo Development

During the breeding season, KU-3nAT produced a small amount of semen (watery semen, <100 µL per male fish) or eggs (<200 eggs per female fish) after artificial induction. To test the fertilization and embryonic development capacity of gametes produced by KU-3nAT, five crossing groups were established (Figure 6).

The control group (2nRCC self-cross) exhibited normal fertilization and embryonic development, progressing through zygote, 1-cell, morula, blastula, gastrula, tail bud, and somite stages, and eventually hatching into fry. The self-crossed KU-3nAT failed to undergo normal embryonic development and showed prominent developmental abnormalities during the gastrula stage. Similarly, embryos from the KU-3nAT ♀ × 2nRCC ♂ and 2nRCC ♀ × KU-3nAT ♂ crosses exhibited abnormal development and could not form fry. In contrast, a very few embryos (hatching rate ~10%) from the KU-3nAT ♀ × 4nAT ♂ cross developed normally and produced fry.

## 4. Discussion

### 4.1. Synapsis Failure Activates the Pachytene Checkpoint in 3nAT

Triploid fish typically exhibit gonadal sterility, yet the molecular mechanisms linking chromosome set imbalance to germ cell elimination remain poorly defined. In hybrid animals, when homologous synapsis and recombination are disrupted, the pachytene checkpoint activates downstream apoptotic factors, leading to germ cell apoptosis and reproductive failure [25]. The pachytene checkpoint is a meiosis-specific surveillance mechanism that monitors homologous chromosome recombination and synapsis during meiotic prophase I [23]; when defects remain uncorrected, the checkpoint arrests the cell cycle at pachytene and triggers downstream responses.

In 3nAT, which contains two sets of crucian carp chromosomes and one set of common carp chromosomes, the single set of common carp chromosomes cannot complete synaptonemal pairing during meiotic prophase I. Our TUNEL results confirmed massive apoptosis in untreated 3nAT gonads, while apoptotic signals were markedly reduced after KU55933 treatment, supporting the hypothesis that the pachytene checkpoint is activated in 3nAT due to synapsis failure and subsequently induces germ cell apoptosis. Consistent with this model, cytological analysis of a gynogenetic allodiploid hybrid fish revealed that the synaptonemal complex can form between heterologous chromosomes but frequently exhibits pairing abnormalities and incomplete synapsis [29], providing direct evidence that hybrid fish germ cells face intrinsic meiotic challenges at the chromosomal level.

Downstream responses to pachytene checkpoint activation vary across species, ranging from cell cycle delay in yeast [30,31] to p53-mediated apoptosis in mammals [24]. The finding that 3nAT germ cells undergo ATM/p53-mediated apoptosis upon synapsis failure indicates that fish employ a mammalian-like apoptotic pathway, consistent with the evolutionary conservation of this surveillance mechanism across vertebrates.

This finding aligns with observations in other triploid fish species. In sterile triploid crucian carp (3nCC), chromosomes exhibit unipolar radial distribution with partial pairing, and TUNEL assays revealed massive spermatocyte apoptosis [32]. In triploid dojo loach, asynapsis and irregular synapsis of heterologous chromosomes are the primary causes of male sterility [33]. In triploid yellow drum, germ cells arrest at abnormal metaphase I [34]. These convergent findings suggest that synapsis failure-induced germ cell apoptosis represents a common sterility mechanism in triploid fish, although the specific molecular signaling pathways had not been elucidated prior to the present study. More broadly, ploidy level has been proposed as a key determinant of gametogenic pathway choice in fish hybrids, with different ploidy levels triggering distinct reproductive strategies [35]. In this context, the apoptotic elimination of 3nAT germ cells can be understood as one specific outcome of a ploidy-dependent regulatory network.

Together, these findings support a model in which meiotic abnormalities associated with defective chromosome pairing are linked to ATM/p53-associated germ-cell apoptosis in 3nAT.

### 4.2. ATM/p53-Mediated Apoptosis as the Downstream Executor

In this study, our pharmacological data are consistent with the involvement of ATM/p53-associated signaling in the sterility of allotriploid crucian carp. Under normal conditions, DSBs induced by SPO11 during meiotic prophase I in 3nAT germ cells cannot be repaired through homologous recombination due to the absence of homologous pairing partners for the common carp-derived chromosomes. ATM is activated in response to persistent DSBs, and the downstream p53 protein is phosphorylated, inducing germ cell apoptosis and resulting in gonadal sterility. The marked reduction in TUNEL signals and downregulation of pro-apoptotic genes (*p53*, *bad*, *bid*) upon KU55933 treatment are consistent with ATM/p53-associated signaling acting downstream of the proposed pachytene checkpoint in 3nAT. This is mechanistically distinct from the neuroendocrine rescue strategy previously attempted [22], which targets hormonal pathways downstream of gamete maturation without addressing the meiotic arrest itself. The fact that blocking a single kinase can partially rescue meiotic progression indicates that the arrest is not due to an intrinsic inability of germ cells to initiate meiotic programs, but rather to active ATM/p53-mediated surveillance that can be pharmacologically lifted. Without ATM/p53-mediated apoptosis, germ cells arrested at the pachytene stage survived and showed altered transcript abundance of meiosis-related genes, as evidenced by the upregulation of meiosis-related genes (*spo11*, *rad51*, *dmc1*, *rec8*, *scp1*) in KU-3nAT gonads.

At the molecular level, p53 functions as a dose-dependent executor of the DNA damage response: low-level damage triggers cell cycle arrest for repair, whereas persistent damage induces apoptosis through transcriptional activation of pro-apoptotic factors and mitochondrial apoptotic cascades [6,7,8]. This dose-dependent logic may explain why 3nAT germ cells, which accumulate persistent unrepaired DSBs due to synapsis failure, are preferentially directed toward apoptosis. Additionally, ATM controls meiotic DSB numbers through a negative feedback loop—suppressing further SPO11-dependent DSB formation upon activation—and ATM deficiency dramatically increases DSB numbers [36,37].

The role of ATM/p53-mediated apoptosis at the pachytene checkpoint is documented in mammals and supported by a medaka study. In mouse spermatocytes, ATM-dependent signaling enforces recombination-dependent pachytene arrest, and ATM deficiency allows cells to bypass this checkpoint [38]. Downstream, p53 and TAp63 serve as critical effectors of the MRE11-ATM-CHK2 pathway, and p53 also controls meiotic crossover formation under normal conditions [39,40]. In *Dmc1*-knockout medaka, defective synapsis leads to massive testicular apoptosis accompanied by p53 and caspase-9 upregulation [41]. Together with our results, these findings are consistent with possible involvement of ATM/p53-associated apoptosis in responses to pachytene-stage defects across some vertebrate taxa, though broad evolutionary conservation cannot be concluded solely from current evidence. Our study provides pharmacological evidence in an allotriploid fish that synapsis-related meiotic abnormalities are associated with this pathway. Consistently, declining ATM function in mammalian oocytes leads to meiotic defects and compromised gamete quality [42], providing convergent evidence from a physiological model that ATM functional integrity is essential for gamete quality across vertebrates.

### 4.3. KU55933 Promotes Meiotic Progression and Gamete Production

After KU55933 treatment, the gonadal development of 3nAT was significantly improved. In the ovaries, KU-3nAT produced more phase II oocytes compared to the undeveloped ovaries of untreated 3nAT. In the testes, cavity structures with sperm-like cells were observed. The upregulation of sperm maturation-related genes (*spef2*, *rnf8*, *ift27*, *tekt1*, *piwil2*) and the vitellogenesis gene (*vtg*) in KU-3nAT is consistent with the idea that KU55933 treatment supports gamete-related developmental programs at the molecular level. These results are consistent with a previous study in which sex hormone rescue promoted gamete maturation-related gene expression in 3nAT [22], but the mechanism is fundamentally different: sex hormone rescue targets the neuroendocrine pathway, whereas KU55933 directly targets the meiotic pachytene checkpoint.

The specificity and efficacy of KU55933 as an ATM inhibitor have been well documented in various cellular contexts. In U2OS cells with ionizing radiation-induced DSBs, KU55933 treatment inhibited p53 phosphorylation [26]. KU55933 treatment also decreased the expression and phosphorylation of ATM target proteins H2AX and p53 [27]. Furthermore, KU55933 treatment inhibited the activation of the ATM/p53 pathway, decreased autophagy, and alleviated the toxicity of microcystin LR to mouse spermatogonial cell line GC-1 cells [28]. In porcine oocytes, KU55933 treatment blocked meiotic maturation, reducing germinal vesicle breakdown and first polar body extrusion [43], suggesting that ATM activity is required for normal meiotic progression in oocytes. These findings are consistent with our observation that KU55933 effectively inhibited ATM function, thereby blocking the pachytene checkpoint-mediated apoptotic pathway and permitting partial meiotic-related developmental progression.

This distinction is significant: the meiotic arrest in 3nAT results not from an intrinsic inability of germ cells to initiate meiotic programs, but from active suppression of meiotic progression by ATM/p53-mediated surveillance. If this surveillance-linked apoptotic response is attenuated, the intrinsic meiotic machinery may regain partial developmental competence.

This is consistent with findings in porcine oocytes, where KU55933 treatment affected meiotic maturation by modulating ATM-dependent DNA damage signaling and cell cycle gene expression [43]. However, it is important to note that while KU55933 treatment enabled meiotic progression, the quality of the resulting gametes was severely compromised, highlighting the dual role of the ATM/p53 checkpoint: it eliminates defective germ cells but also prevents the production of potentially harmful aneuploid gametes.

The fact that KU55933 treatment enabled 3nAT to produce gametes indicates that the meiotic arrest can be partially bypassed when ATM/p53-mediated apoptosis is blocked. However, chromosome segregation during meiosis in KU-3nAT is likely disrupted, because the single set of common carp chromosomes cannot pair properly and will segregate randomly.

### 4.4. Abnormal Sperm Structure and Embryonic Lethality

The sperm produced by KU-3nAT exhibited severe structural abnormalities, including rounded heads with retained cytoplasm, absence or malformation of flagella, and irregular chromatin condensation. SEM and TEM observations further revealed folds on the sperm head surface, chromatin of varying condensation states, and the presence of apoptotic bodies. These abnormalities suggest that although KU55933 treatment allowed germ cells to bypass the pachytene checkpoint and continue meiosis, the subsequent spermiogenesis process—including cytoplasm removal, chromatin condensation, and flagellar assembly—was defective. It is hypothesized that aneuploid chromosome composition arising from random segregation of unpaired common carp chromosomes could disturb the expression of genes required for spermiogenesis, which may account for the observed structural defects.

The fertilization and embryo development experiments demonstrated that the gametes produced by KU-3nAT had limited fertilization ability and lost the capacity for normal embryonic development. Self-crossed KU-3nAT embryos and hybrid embryos between KU-3nAT and 2nRCC arrested after the gastrula stage. This developmental failure may be associated with abnormal or aneuploid chromosome complements in the gametes, although we have not directly examined gamete or embryonic chromosome constitution in the present study. On cytogenetic grounds, random segregation of the common-carp-derived chromosomes during meiosis I would be expected to produce gametes with unbalanced chromosome complements, a condition that could underlie the observed embryonic lethality. The few fry produced by the KU-3nAT ♀ × 4nAT ♂ cross raise the possibility of androgenesis, whereby the paternal genome from 4nAT sperm alone supports embryonic development. However, this interpretation remains speculative without direct ploidy analysis or genotyping of the offspring, which should be pursued in future studies.

The production of aneuploid gametes by triploid hybrids has been documented in other fish species. In triploid Chinese loach hybrids between tetraploid and diploid populations, both males and females generated gametes, but progenies did not survive beyond larval stages, with modal ploidy levels of approximately 1.5n in gametes and 2.5n in progeny [44]. Similarly, in triploid yellow drum, gametogenesis was severely impaired with no mature gametes produced [34]. These findings across different triploid fish species consistently demonstrate that aneuploid gametes resulting from chaotic chromosome segregation during triploid meiosis are incapable of supporting normal embryonic development, reinforcing the conclusion that the fundamental cause of triploid sterility lies in the inability to properly segregate heterologous chromosome sets. Future studies employing single-cell sequencing of KU-3nAT sperm may help identify the specific aneuploidy-sensitive genes responsible for defective spermiogenesis and flagellar assembly. More direct approaches including ploidy measurement, genotyping and chromosome-segregation cytology should also be prioritized to test the above-mentioned hypotheses.

It should be noted that activation of the ATM/p53 apoptotic pathway reported here is mainly inferred from qPCR transcript quantification of pathway-associated genes together with TUNEL-detected gonadal apoptotic signals, and direct protein-level supporting evidence remains absent. While commercial p53 antibodies validated for zebrafish are available, no antibodies targeting ATM, phosphorylated ATM, or phosphorylated p53 have been experimentally validated for allotriploid crucian carp. This prevents us from performing Western blot or immunofluorescence to examine the activating phosphorylation of ATM and p53. Accordingly, we cannot fully rule out the potential contributions of other parallel DNA-damage-responsive cascades to germ-cell apoptosis. Future protein-based functional assays, including caspase-3 activity measurement or immunodetection of phosphorylated ATM/p53 with carp-validated reagents, are needed to definitively establish the role of the ATM/p53 signaling axis in pachytene-checkpoint-mediated germ-cell clearance in polyploid fish.

## 5. Conclusions

In summary, our results support a model in which meiotic chromosome-pairing abnormalities in 3nAT are associated with ATM/p53-dependent germ cell apoptosis, while pharmacological inhibition of ATM partially reduces apoptosis and permits limited gamete production; however, the resulting gametes exhibited severe structural abnormalities and limited developmental competence, potentially reflecting abnormal chromosome segregation. Taken together, chromosomal pairing and segregation defects during meiosis are likely to contribute to the sterility of 3nAT, and ATM/p53-associated apoptosis appears to contribute to the elimination of germ cells with meiotic abnormalities. KU55933 relaxes this clearance, allowing defective germ cells to survive and progress, but the resulting gametes remain structurally and functionally abnormal. This study provides evidence that ATM/p53-associated apoptosis contributes to meiotic germ cell elimination in an allotriploid fish with meiotic pairing abnormalities (Figure 7), establishing a mechanistic link between homologous pairing disorder and germ cell apoptosis that advances our understanding of distant hybridization in fish.

The involvement of ATM/p53 signaling in germ cell elimination during triploid meiosis has both evolutionary and applied implications. Although triploid sterility appears to be maintained by active surveillance rather than passive developmental failure alone, the severe gamete abnormalities and extremely limited reproductive success after KU55933 treatment indicate that checkpoint modulation does not restore functional fertility, and strategies to overcome sterility in polyploid fish should therefore be viewed with caution. The partial rescue of gamete production by ATM inhibition further highlights both the potential and inherent limitations of single-checkpoint modulation. Elucidating the precise signaling cascade linking ATM activation to ATM/p53-associated apoptosis during fish meiosis, and exploring whether modulation of additional checkpoint-related pathways can further improve gamete production or developmental competence, remain important directions for future study.

## Figures and Tables

**Figure 1:**
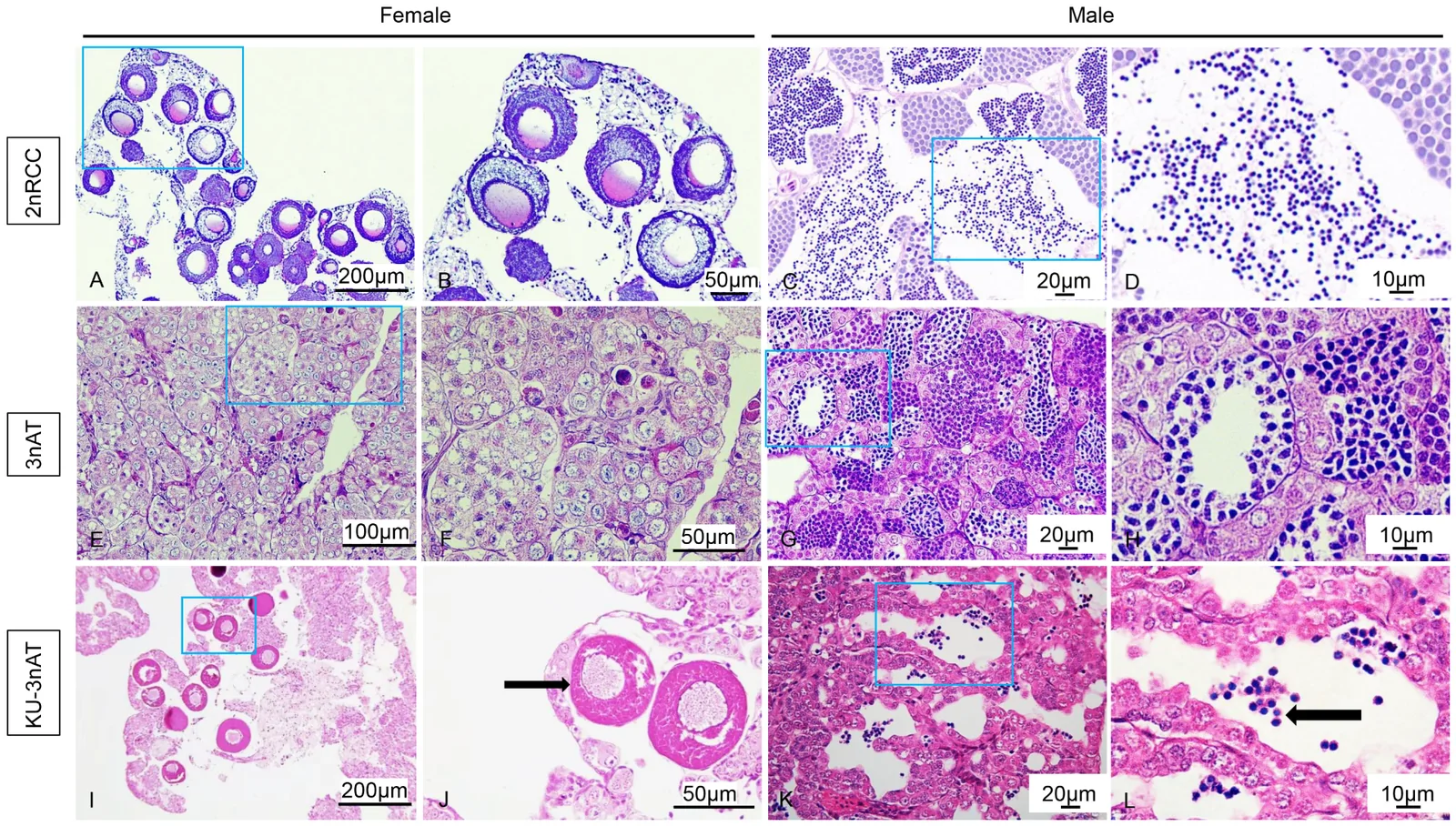
Gonad microstructure of 2nRCC, 3nAT, and KU-3nAT. (**A**,**B**) 2nRCC ovaries contained many growing phase II oocytes; (**E**,**F**) 3nAT ovaries lacked apparent phase II oocytes; (**I**,**J**) KU-3nAT ovaries contained some phase II oocytes. (**C**,**D**) 2nRCC testes contained a large number of mature sperm; (**G**,**H**) 3nAT sperm cells showed abnormal nuclear condensation; (**K**,**L**) KU-3nAT testes contained cavity structures with some sperm.

**Figure 2:**
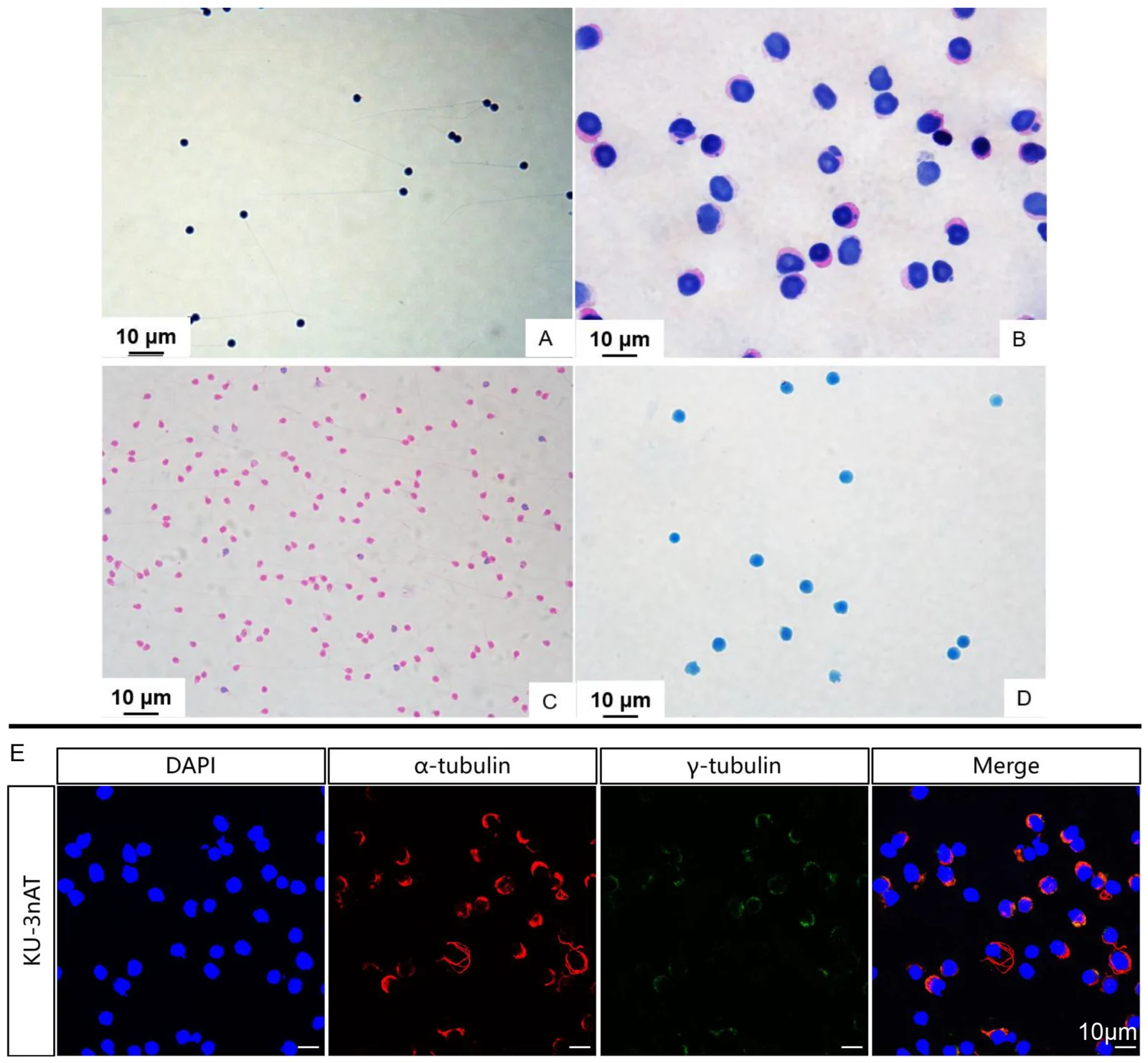
Sperm structure of 2nRCC and KU-3nAT. (**A**) Papanicolaou staining of 2nRCC sperm showing normal head, neck, and flagellum. (**B**) Papanicolaou staining of KU-3nAT sperm showing round head with retained cytoplasm and absent flagellum. (**C**) Aniline blue-eosin staining of 2nRCC sperm (rose-pink mature). (**D**) Aniline blue-eosin staining of KU-3nAT sperm (blue, immature). (**E**) Immunofluorescence of KU-3nAT sperm showing abnormal flagella and neck structure. α-tubulin (red), γ-tubulin (green), DAPI (blue).

**Figure 3:**
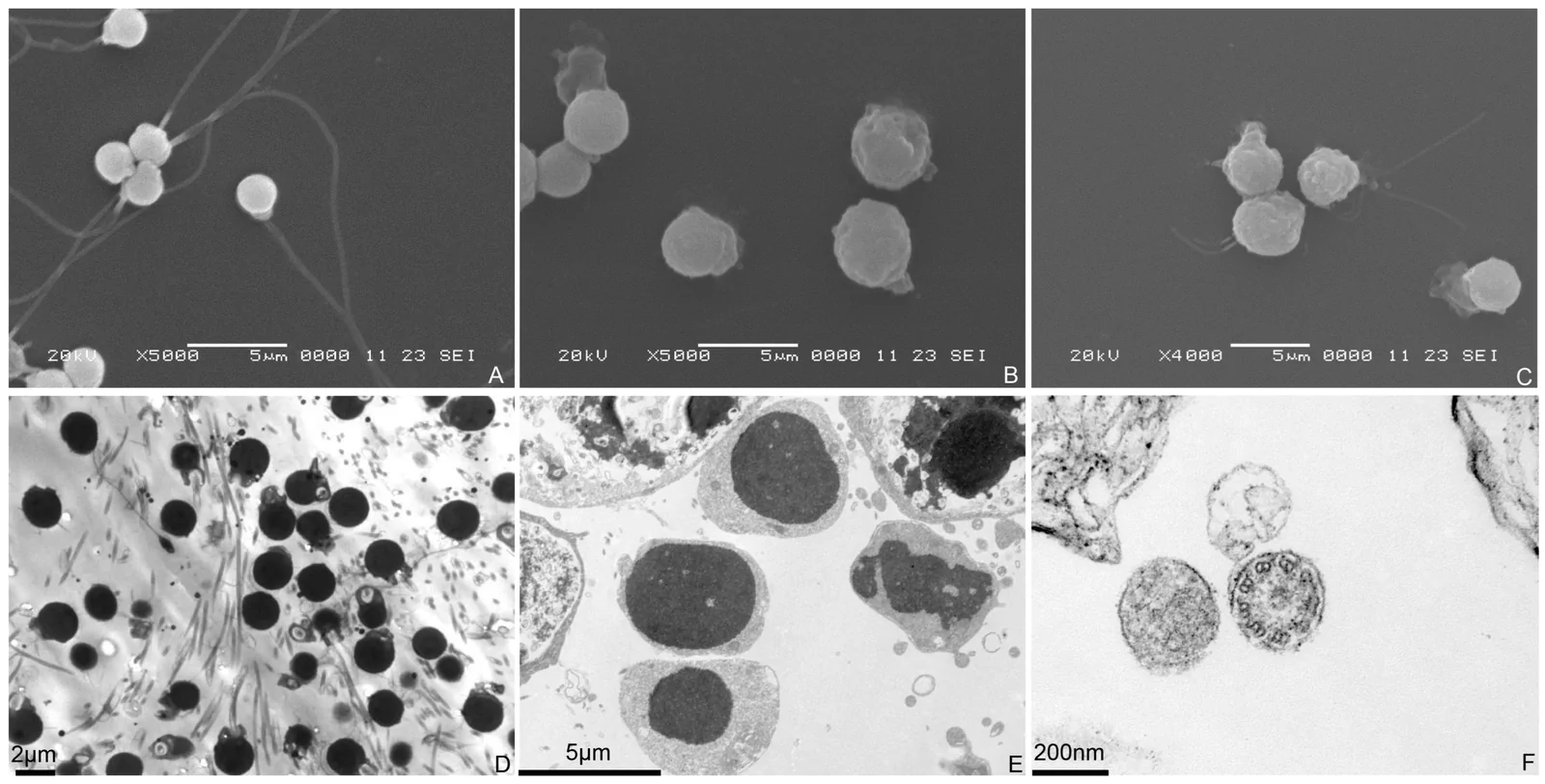
Ultrastructure of KU-3nAT sperm. (**A**) SEM of 2nRCC sperm showing normal morphology with smooth round head, intact neck, and flagellum. (**B**) SEM of KU-3nAT sperm showing folds on the head surface. (**C**) SEM of KU-3nAT sperm with multiple flagella. (**D**) TEM of KU-3nAT sperm head with condensed chromatin of varying sizes. (**E**) TEM of KU-3nAT sperm nucleus forming apoptotic body. (**F**) TEM showing the 9 + 2 axonemal structure of flagellum in KU-3nAT sperm.

**Figure 4:**
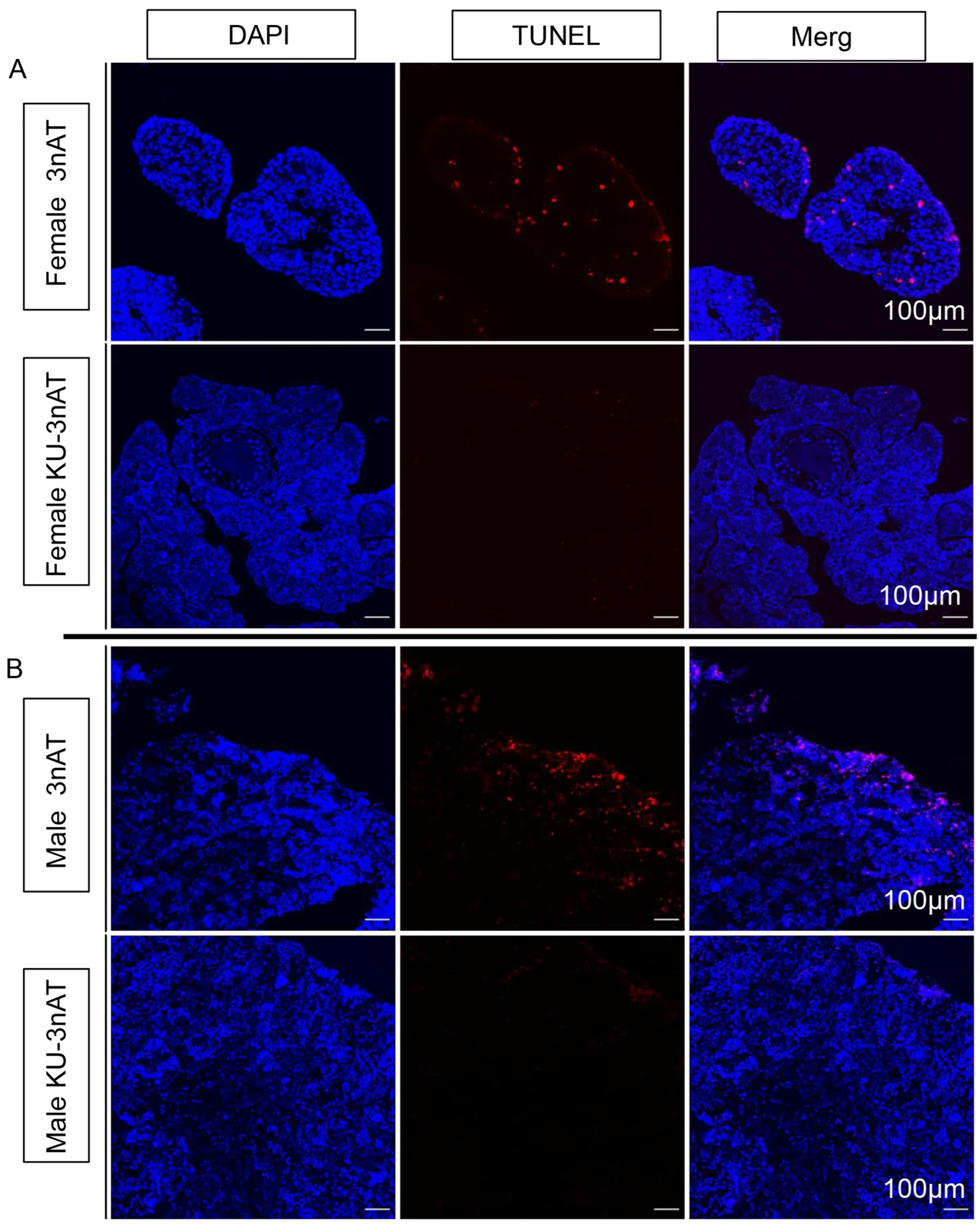
TUNEL assay of germ cell apoptosis in gonads. TUNEL staining (red) of ovarian (**A**) and testicular (**B**) tissues from 3nAT and KU-3nAT. Nuclei were counterstained with DAPI (blue).

**Figure 5:**
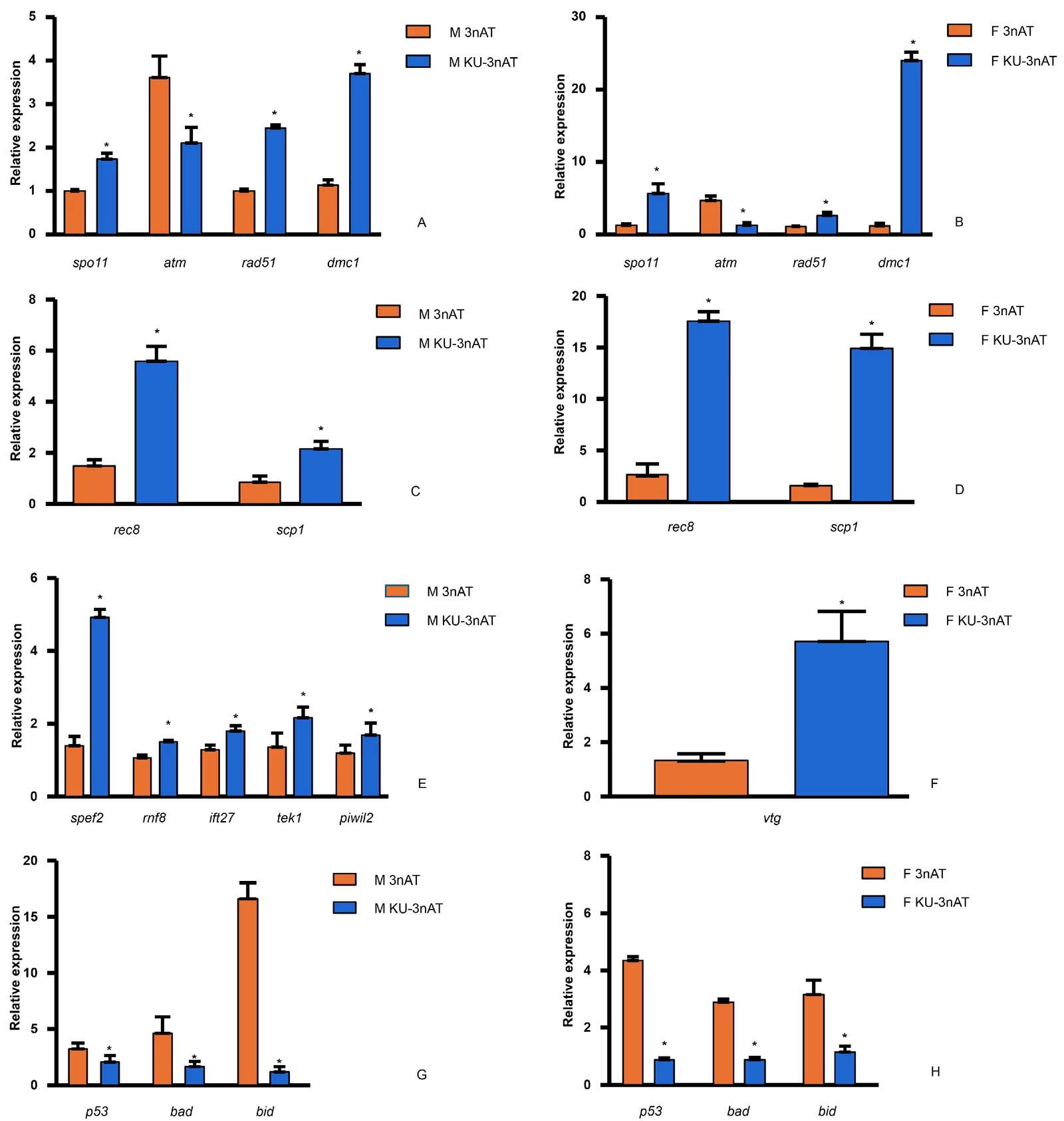
qPCR analysis of gene expression after KU55933 treatment. (**A**–**D**) Meiosis-related genes (*spo11*, *atm*, *rad51*, *dmc1*, *rec8*, *scp1*) in ovaries and testes of 3nAT and KU-3nAT. (**E**) Sperm maturation-related genes (*spef2*, *rnf8*, *ift27*, *tekt1*, *piwil2*) in testes. (**F**) Vitellogenesis gene (*vtg*) in female liver. (**G**,**H**) Pro-apoptotic genes (*p53*, *bad*, *bid*) in testes (**G**) and ovaries (**H**). All data are mean ± S.E.M. (*n* = 3). * represents statistically significant difference *p* < 0.05 versus 3nAT.

**Figure 6:**
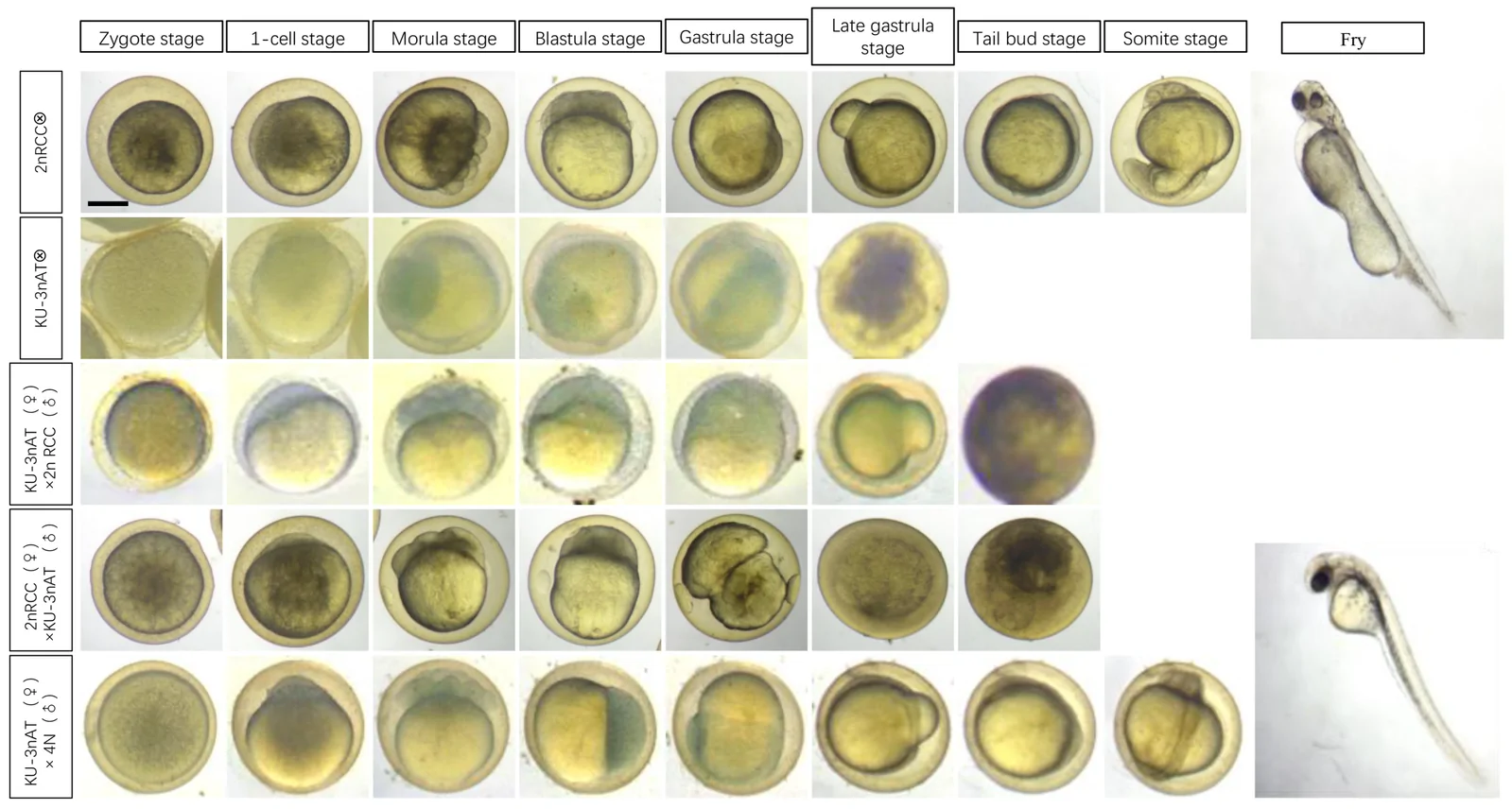
Embryonic development of different crossing groups. Representative images of embryonic development from five crossing groups at the indicated stages: 2nRCC ♀ × 2nRCC ♂ (control), KU-3nAT ♀ × KU-3nAT ♂, KU-3nAT ♀ × 2nRCC ♂, 2nRCC ♀ × KU-3nAT ♂, and KU-3nAT ♀ × 4nAT ♂. Scale bars: 0.5 mm.

**Figure 7:**
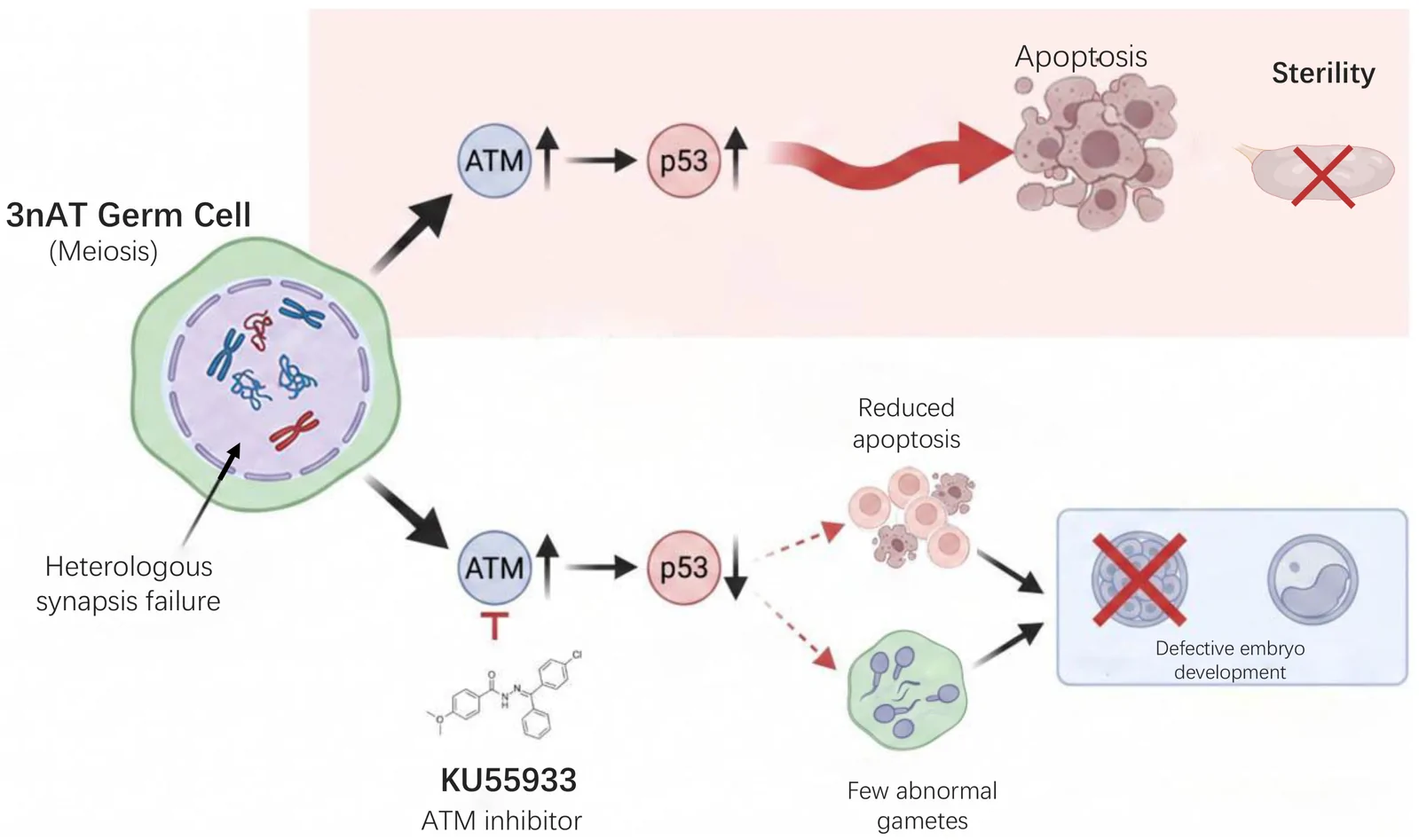
Proposed mechanistic model of ATM-p53 signaling in the sterility of 3nAT. Solid arrows indicate activation or promotion; upward arrows denote increased activity/expression; the red T-bar represents inhibition of ATM by KU55933; dashed arrows indicate partial or attenuated effects.

**Table 1:** Primer sequences used for quantitative real-time PCR (qPCR) in this study.

Gene	Forward Primer	Reverse Primer
*spo11*	CTTCCAATGTTAATGGGGATCAGA	CAGTTTCCTCACCCATCAGTCTGC
*atm*	ATGGGGATGGGGCATTACAGG	GGTTCATGGTCCAATCAAACAG
*rad51*	CTGACGGAGGCGGCGAAG	AGCAATCTCTCTGGACGGAAGG
*dmc1*	ACTATGTGTAACCGCTCAG	GATGCTCACTCCGTATATGC
*rec8*	CGCCCTTAACATGAAGTCCAACATTG	ATAACCAGTCTCCATAACCGCCAAAG
*scp1*	TCAAAGAGGCAAACAGTCAAAACCAAC	AGCATCTCACCCCTCCCCTTGTTC
*spef2*	ACGGTAAGCCCGCTGTATG	CTCGGGTGTAGTCTTGATTGTTTG
*rnf8*	TCTGTGAGGAGGAACGACG	ATCCTTCCCGAGCGTGTT
*ift27*	GGGGAATAAGAGTGACCTG	GCGAGGCTCTGGGATGA
*tekt1*	AAATCTACCAAAACACACGCAACAG	CACCTCCTTCTCCTTCAACAGC
*piwil2*	AGGAGTGCGATTACCGACAG	TGCCGCTCAGGCATCTTT
*vtg*	GTTCCCACTTGTTCTTCTG	GCATCAACCTGGACTCTAA
*p53*	CCATCCTCACACAATCATCAC	TCTGAAGTTGCTCTCCTC
*bad*	GGGCGTGTGCGGGGTCTATTC	AGCAGCAGGAGCAGATTGAGAAC
*bid*	GCGGATGGACACAGTTTCTCAG	CTTGGGACAGGACGCTTTGC
*β-actin*	GCCCTGCCCCATGCCATCCT	AGTGCCCATCTCCTGCTCGA

## Data Availability

The original contributions presented in this study are included in the article. Further inquiries can be directed to the corresponding author.

## References

[1] Falck J., Coates J., Jackson S.P. (2005). Conserved modes of recruitment of ATM, ATR and DNA-PKcs to sites of DNA damage. Nature.

[2] Lee J.H., Paull T.T. (2005). ATM activation by DNA double-strand breaks through the Mre11-Rad50-Nbs1 complex. Science.

[3] Jazayeri A., Falck J., Lukas C., Bartek J., Smith G.C.M., Lukas J., Jackson S.P. (2006). ATM- and cell cycle-dependent regulation of ATR in response to DNA double-strand breaks. Nat. Cell Biol..

[4] Saito S., Goodarzi A.A., Higashimoto Y., Noda Y., Lees-Miller S.P., Appella E., Anderson C.W. (2002). ATM mediates phosphorylation at multiple p53 sites, including Ser46, in response to ionizing radiation. J. Biol. Chem..

[5] Yoshiyama K.O., Sakaguchi K., Kimura S. (2013). DNA damage response in plants: Conserved and variable response compared to animals. Biology.

[6] Li G., Ho V.C. (1998). p53-Dependent DNA repair and apoptosis respond differently to high- and low-dose ultraviolet radiation. Br. J. Dermatol..

[7] Agarwal M.L. (1998). The p53 network. J. Biol. Chem..

[8] Nii T., Marumoto T., Tani K. (2012). Roles of p53 in various biological aspects of hematopoietic stem cells. J. Biomed. Biotechnol..

[9] Tiwary B.K., Kirubagaran R., Ray A.K. (2000). Gonadal development in triploid *Heteropneustes fossilis*. J. Fish Biol..

[10] Felip A., Piferrer F., Carrillo M., Zanuy S. (2001). Comparison of the gonadal development and plasma levels of sex steroid hormones in diploid and triploid sea bass, *Dicentrarchus labrax* L.. J. Exp. Zool..

[11] Haffray P., Bruant J.S., Facqueur J.M., Fostier A. (2005). Gonad development, growth, survival and quality traits in triploids of the protandrous hermaphrodite gilthead seabream *Sparus aurata* (L.). Aquaculture.

[12] Manning A.J., Burton M.P.M., Crim L.W. (2004). Reproductive evaluation of triploid yellowtail flounder, *Limanda ferruginea* (Storer). Aquaculture.

[13] Zhu L., He W., Zhang H., Sun Y., Li Q., Zhou L., Zhu S., Tao M., Zhou Y., Zhao R. (2021). Unconventional meiotic process of spermatocytes in male *Cyprinus carpio*. Reprod. Breed..

[14] Long Y., Liu S., Huang W., Zhang J., Sun Y., Zhang C., Chen J., Liu Y. (2006). Comparative studies on histological and endocrinological changes during gonadal development between diploid and triploid female red crucian carp. Cell Tissue Res..

[15] Long Y., Tao M., Liu S., Huang W., Zhang J., Sun Y., Zhang C., Chen J., Liu Y. (2009). Differential expression of Gnrh2, Gthβ, and Gthr genes in sterile triploids and fertile tetraploids. Cell Tissue Res..

[16] Xu K., Wen M., Duan W., Ren L., Hu F., Xiao J., Wang J., Tao M., Zhang C., Wang J. (2015). Comparative analysis of testis transcriptomes from triploid and fertile diploid cyprinid fish. Biol. Reprod..

[17] Li W., Tan H., Liu J., Hu J., Cui J., Wang S., Liu Q., Hu F., Ren L., Tao M. (2018). Comparative analysis of testis transcriptomes associated with male infertility in triploid cyprinid fish. Reprod. Fertil. Dev..

[18] Liu S., Cao Y., He X., Li J., Yun L. (2001). The formation of tetraploid hybrids of common carp with red crucian carp and the evolutionary significance of tetraploidization in vertebrate. Eng. Sci..

[19] Chen S., Wang J., Liu S., Qin Q., Xiao J., Duan W., Luo K., Liu J., Liu Y. (2009). Biological characteristics of an improved triploid crucian carp. Sci. China Ser. C Life Sci..

[20] Liu S., Liu Y., Zhou G., Zhang X., Luo C., Feng H., He X., Zhu G., Yang H. (2000). The formation of tetraploid stocks of red crucian carp × common carp hybrids as an effect of interspecific hybridization. Aquaculture.

[21] Cui J., Luo M., Gao X., Zhang H., Zhang X., Ren L., Liu S. (2022). The formation and biological characterization of two allotriploid fish derived from interploid crosses. Reprod. Breed..

[22] Wang J., Wang W., Li J., Luo Z., Li Z., Chai M., Xiang C., Luo K., Zhao R., Liu S. (2022). Effects of sex hormone rescue on gametogenesis in allotriploid crucian carp. Aquaculture.

[23] Roeder G.S., Bailis J.M. (2000). The pachytene checkpoint. Trends Genet..

[24] Longhese M.P., Bonetti D., Guerini I., Manfrini N., Clerici M. (2009). DNA double-strand breaks in meiosis: Checking their formation, processing and repair. DNA Repair.

[25] Li X.C., Barringer B.C., Barbash D.A. (2009). The pachytene checkpoint and its relationship to evolutionary patterns of polyploidization and hybrid sterility. Heredity.

[26] Hickson I., Zhao Y., Richardson C.J., Green S.J., Martin N.M.B., Orr A.I., Reaper P.M., Jackson S.P., Curtin N.J., Smith G.C.M. (2004). Identification and characterization of a novel and specific inhibitor of the ataxia-telangiectasia mutated kinase ATM. Cancer Res..

[27] Schweikl H., Petzel C., Bolay C., Hiller K.A., Buchalla W., Krifka S. (2014). 2-Hydroxyethyl methacrylate-induced apoptosis through the ATM- and p53-dependent intrinsic mitochondrial pathway. Biomaterials.

[28] Tian Z., Liu H., Chen X., Losiewicz M.D., Wang R., Du X., Wang B., Ma Y., Zhang S., Shi L. (2021). The activated ATM/p53 pathway promotes autophagy in response to oxidative stress-mediated DNA damage induced by Microcystin-LR in male germ cells. Ecotoxicol. Environ. Saf..

[29] Wang J., Wang W., Li J., Zhang Y., Luo K., Han L., Xiang C., Chai M., Luo Z., Zhao R. (2023). Formation of the synaptonemal complex in a gynogenetic allodiploid hybrid fish. Front. Genet..

[30] Perez-Hidalgo L., Moreno S., San-Segundo P.A. (2008). The fission yeast meiotic checkpoint kinase Mek1 regulates nuclear localization of Cdc25 by phosphorylation. Cell Cycle.

[31] Sourirajan A., Lichten M. (2008). Polo-like kinase Cdc5 drives exit from pachytene during budding yeast meiosis. Genes Dev..

[32] Zhang C., Li Q., Zhu L., He W., Yang C., Zhang H., Sun Y., Zhou L., Sun Y., Zhu S. (2021). Abnormal meiosis in fertile and sterile triploid cyprinid fish. Sci. China Life Sci..

[33] Kuroda M., Fujimoto T., Murakami M., Yamaha E., Arai K. (2019). Aberrant meiotic configurations cause sterility in clone-origin triploid and inter-group hybrid males of the dojo loach, *Misgurnus anguillicaudatus*. Cytogenet. Genome Res..

[34] Yang Y., Lu L., Chen R., Yu L., Hu W., Xu D. (2023). Production of sterile mono-sex triploid yellow drum (*Nibea albiflora*): Genotypic females and sex-reversed phenotypic males with emphasis on utilization as surrogate broodstock. Fish Physiol. Biochem..

[35] Dedukh D., Marta A., Myung R.-Y., Ko M.-H., Choi D.-S., Won Y.-J., Janko K. (2024). A cyclical switch of gametogenic pathways in hybrids depends on the ploidy level. Commun. Biol..

[36] Lukaszewicz A., Lange J., Keeney S., Jasin M. (2018). Control of meiotic double-strand-break formation by ATM: Local and global views. Cell Cycle.

[37] Kurzbauer M.T., Janisiw M.P., Paulin L.F., Prusen Mota I., Tomanov K., Krsicka O., von Haeseler A., Schubert V., Schlogelhofer P. (2021). ATM controls meiotic DNA double-strand break formation and recombination and affects synaptonemal complex organization in plants. Plant Cell.

[38] Pacheco S., Marcet-Ortega M., Lange J., Jasin M., Keeney S., Roig I. (2015). The ATM signaling cascade promotes recombination-dependent pachytene arrest in mouse spermatocytes. PLoS Genet..

[39] Marcet-Ortega M., Pacheco S., Martinez-Marchal A., Castillo H., Flores E., Jasin M., Keeney S., Roig I. (2017). p53 and TAp63 participate in the recombination-dependent pachytene arrest in mouse spermatocytes. PLoS Genet..

[40] Marcet-Ortega M., Maldonado-Linares A., Lopez-Panades M., Roig I. (2022). p53 controls meiotic prophase progression and crossover formation. Int. J. Mol. Sci..

[41] Chen J., Cui X., Jia S., Luo D., Cao M., Zhang Y., Wang D. (2016). Disruption of dmc1 Produces Abnormal Sperm in Medaka (*Oryzias latipes*). Sci. Rep..

[42] Suzuki R., Sato Y., Kikuchi K., Ota Y., Ito S., Kishida T., Iwata H., Sato-Shimizu E., Ozono K., Sasaki H. (2024). The role of declining ataxia-telangiectasia-mutated (ATM) function in oocyte aging. Cell Death Discov..

[43] Lin Z.L., Kim N.H. (2015). Role of ataxia-telangiectasia mutated (ATM) in porcine oocyte in vitro maturation. Cell Biol. Int..

[44] Li Y.J., Gao Y.C., Zhou H., Ma H.Y., Lin Z.Q., Ma T.Y., Sui Y., Arai K. (2016). Aneuploid progenies of triploid hybrids between diploid and tetraploid loach *Misgurnus anguillicaudatus* in China. Genetica.

